# Data on quantification of PAHs and elemental content in dry *Camellia sinensis* and herbal tea

**DOI:** 10.1016/j.dib.2018.08.181

**Published:** 2018-09-05

**Authors:** Omowunmi H. Fred-Ahmadu, Nsikak U. Benson

**Affiliations:** Analytical and Environmental Chemistry Unit, Department of Chemistry, Covenant University, Km 10 Idiroko Road, Ota, Ogun State, Nigeria

## Abstract

Here we present data on potentially toxic metals and polycyclic aromatic hydrocarbons (PAHs) in commercially sold tea brands in Nigeria. The article provides data on the sequential extraction and the pseudo-total concentrations of eight metals (Cd, Cr, Cu, Mn, Ni, Pb, V and Zn) and polycyclic aromatic hydrocarbons (PAHs) in dry *Camellia sinensis* and herbal tea. The three-step Community Bureau of Reference (BCR) method and acid digestion with aqua regia were adopted for sequential and total metal extractions, respectively. The extraction of branded tea samples for PAHs analysis has been described in “Concentrations, sources and risk characterisation of polycyclic aromatic hydrocarbons (PAHs) in green, herbal and black tea products in Nigeria” [1] and “Polycyclic Aromatic Hydrocarbons (PAHs) Occurrence and Toxicity in *C. sinensis* and Herbal Tea” [2]. Elemental and PAHs analyses of extracts were determined by Microwave Plasma Atomic Emission Spectroscopy (Agilent MP-AES 4100) and Agilent gas chromatograph 7890A coupled with flame ionization detector (FID), respectively.

**Specifications table**

**Table:** 

Subject area	Chemistry
More specific subject area	Analytical Chemistry
Type of data	Tables, figures
How data was acquired	Microwave Plasma Atomic Emission Spectrometer (Agilent 4100 MP-AES), Agilent gas chromatograph 7890A with flame ionization detector (GC-FID).
Data format	Raw data, analysed.
Experimental factors	Tea samples were oven-dried, milled, and later extracted using the Community Bureau of Reference (BCR) method. Samples for pseudo-total analysis for were digested using a mixture of conc. HCl (37%, Sigma Aldrich) and HNO_3_ (70%, BDH) in the ratio 3:1. For PAHs, 0.5 g of each tea sample was extracted using N-hexane.
Experimental features	Elemental content determined using MP-AES. PAHs analysis using GC-FID.
Data source location	Lagos and Ogun state, Nigeria.
Data accessibility	Data included in this article.
Related research article	“Chemical speciation and characterization of trace metals in dry *Camellia sinensis* and herbal tea marketed in Nigeria” (Fred-Ahmadu et al., 2018, *In press*) [3]. “Levels of polycyclic aromatic hydrocarbons (PAH4) in some popular tea brands in Nigeria.” (Benson et al., 2017) [4].

**Value of the data**

• The data provides information on the elemental concentrations of green, herbal and black tea brands commonly consumed in Nigeria.
• The quantification data on US-EPA priority PAHs is provided in some selected tea samples.
• The data could be used in assessing the degree of risks associated with heavy metals and PAHs exposure.
• The acquired fractionation data will be advantageous for the scientific community interested in assessing the mobility and bioavailability of metal species.

## 1 Data

**Fig. 1:**
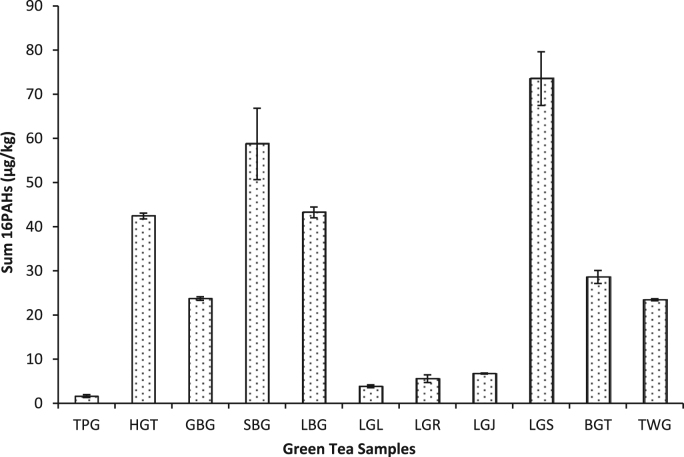
Total PAH Concentrations in branded *Camellia sinensis* (green tea) samples (n = 11).

**Fig. 2:**
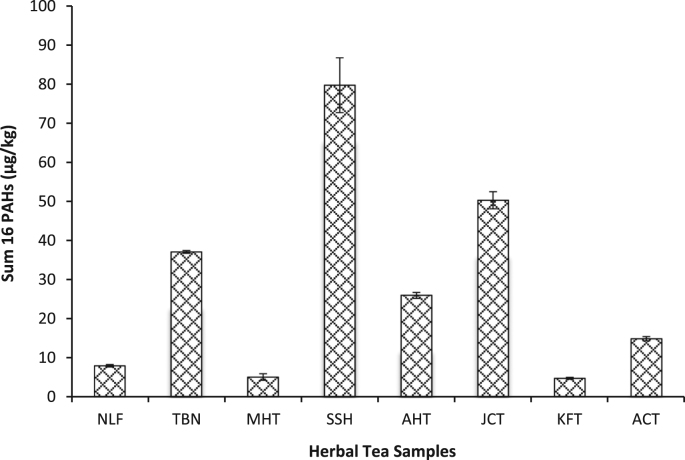
Total PAH Concentrations in branded herbal tea samples (*n* = 8).

**Fig. 3:**
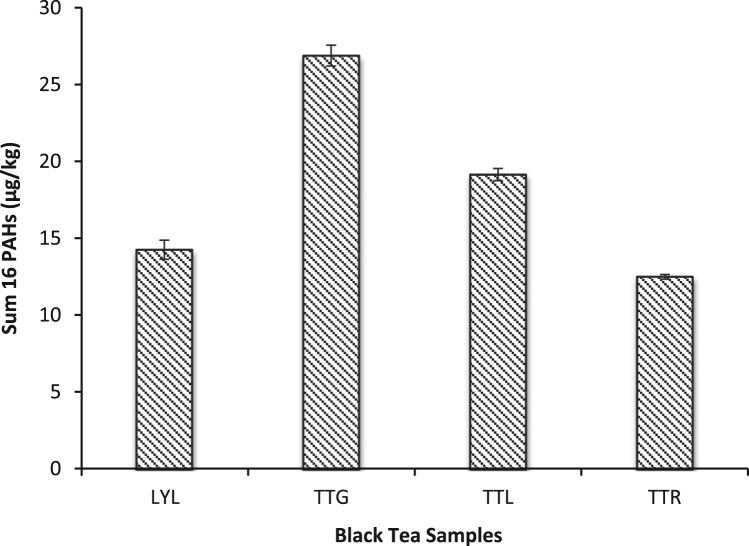
Total PAH Concentrations in branded black tea samples (*n* = 4).

This data article presents fractionation, pseudo-total elemental and PAHs concentrations twenty-three dry tea samples. Table 1 presents the brand names of the tea products and their sample codes adopted for this study. The BCR sequential extraction procedure is showed in Table 2. The mean concentrations of heavy metals in the various fractions (F1, F2, F3 and F4) are represented in Table 3, Table 4, Table 5, respectively. Table 6 shows the mean pseudo-total concentrations. The total PAH concentrations in *Camellia sinensis*, herbal and black tea samples are shown in Fig. 1, Fig. 2, Fig. 3.

**Table 1:** Green, Black and Herbal Tea Samples Used in the Present Study.

**Tea type**	**Product name**	**Sample code**	**Country of origin**
Green Tea	Bigelow Green Tea	BGT	USA
	Gold blend Green Tea	GBG	Sri Lanka
	Heladiv Green Tea	HGT	Sri Lanka
	Lipton Green Tea (*blackberry pomegranate*)	LGB	USA
	Lipton Green Tea (*lemon and ginseng*)	LGL	USA
	Lipton Green Tea (*red goji raspberry*)	LGR	USA
	Lipton Green Tea (*jasmine passion with fruit*)	LGJ	USA
	Lloyd Green Sense (*aloe vera*)	LGS	Poland
	Super blend Green Tea (*vanilla*)	SBG	Sri Lanka
	Twinings Pure Green Tea	TWG	United Kingdom
	Ty-phoo Pure Green Tea	TPG	United Kingdom
	Lipton Yellow Label Tea	LYL	Nigeria
Black Tea	Top Tea (*ginger*)	TTG	Nigeria
	Top Tea (*lime and lemon*)	TTL	Nigeria
	Top Tea (*regular*)	TTR	Nigeria
Herbal Tea	Anti-Cancer Tea	ACT	China
	Anti-hypertensive Tea	AHT	China
	Joint Care Tea	JCT	China
	Kidney Flush Tea	KFT	China
	Moringa Herbal Tea	MHT	Nigeria
	Natural Liver Flush Tea	NLF	China
	Sahul Slim Herbal Tea	SSH	India
	Tranquilizing & Brain Nourishing Tea	TBN	China

**Table 2:** Detailed sequential extraction procedure and analytical reagents used.

**Fraction**	**Code**	**Extraction Procedure**
Soluble/Exchangeable fraction/Bound to carbonates	F1	1.0 g of oven-dried sample was leached with 20 mL of 0.1 mol/L CH_3_COOH following agitation using Stuart orbital shaker SSL1 at 300 rpm for 16 h. Mixture was centrifuged at 4000 rpm for 20 mins, extract decanted and filtered using Whatman® filter paper. Residue was washed several times with distilled water, centrifuged at 3000 rpm for 10 mins.
Reducible – associated with oxides of Fe & Mn	F2	Residue from F1 was extracted with 20 mL of NH_4_OH.HCl and later agitated using a shaker at 300 rpm for 16 h. Mixture was centrifuged at 4000 rpm for 20 mins, decanted and filtered. Residue was washed with distilled water, centrifuged at 3000 rpm for 10 mins.
Oxidizable – bound to organic matter	F3	Residue from F2 was dispersed in 5 mL of 30% H_2_O_2_ and digested at room temp for 1 hr with occasional shaking. Another aliquot of 5 mL 30% H_2_O_2_ was added and digested at 85 °C in a water bath for 1 hr. Mixture was evaporated on hot plate to about 2 mL and 25 mL 1.0 mol/L NH_4_CH_3_CO_2_ was added. Mixture was centrifuged at 4000 rpm for 20 mins and later decanted and filtered.
Residual	F4	Residue F3 + 10 mL (conc. HNO_3_, HCl and HClO_4_) (6:2:5) and agitated at 300 rpm for 5 h at 80 °C using Thermo Scientific maxQ 4000 shaker. Another aliquot of 2 mL of the acid mix was added and transferred to a hot plate at temp between 150 - 200 °C.

**Table 3:** Concentration (mean ± S.D) (mg/kg) of Metals from Sequential Extraction of Herbal Tea Samples (n = 8).

	F1	F2	F3	F4		F1	F2	F3	F4
**NLF**					**AHT**				
Cd	0.66 ± 0.03	0.17 ± 0.07	1.61 ± 0.01	0.64 ± 0.02	Cd	0.68 ± 0.03	0.20 ± 0.17	1.53 ± 0.01	0.41 ± 0.04
Cr	0.36 ± 0.01	0.05 ± 0.02	0.902 ± 0.003	0.68 ± 0.01	Cr	0.313 ± 0.001	0.040 ± 0.004	0.881 ± 0.002	0.963 ± 0.004
Cu	0.55 ± 0.02	0.16 ± 0.03	0.54 ± 0.01	0.37 ± 0.02	Cu	0.48 ± 0.02	0.25 ± 0.03	0.51 ± 0.01	0.41 ± 0.03
Mn	16.70 ± 0.01	10.60 ± 0.01	3.72 ± 0.02	1.58 ± 0.04	Mn	20.40 ± 0.01	15.501 ± 0.002	4.59 ± 0.01	0.89 ± 0.01
Ni	0.54 ± 0.01	0.02 ± 0.08	1.86 ± 0.01	0.65 ± 0.001	Ni	0.47 ± 0.01	BDL	1.73 ± 0.01	0.442 ± 0.003
Pb	0.19 ± 0.03	0.08 ± 0.05	0.610 ± 0.004	0.81 ± 0.02	Pb	0.20 ± 0.04	0.06 ± 0.07	0.51 ± 0.02	0.16 ± 0.02
V	0.18 ± 0.04	0.11 ± 0.04	0.02 ± 0.05	0.33 ± 0.04	V	0.16 ± 0.04	0.14 ± 0.01	0.02 ± 0.01	0.21 ± 0.02
Zn	0.52 ± 0.01	0.46 ± 0.02	0.47 ± 0.01	6.15 ± 0.02	Zn	0.58 ± 0.003	0.41 ± 0.02	1.04 ± 0.01	0.21 ± 0.02
**TBN**					**JCT**				
Cd	0.37 ± 0.02	0.54 ± 0.05	1.74 ± 0.03	0.70 ± 0.03	Cd	0.71 ± 0.05	0.21 ± 0.02	1.63 ± 0.02	0.24 ± 0.06
Cr	0.15 ± 0.01	BDL	0.841 ± 0.004	0.67 ± 0.01	Cr	0.32 ± 0.01	0.03 ± 0.04	0.92 ± 0.01	0.960 ± 0.002
Cu	0.36 ± 0.05	0.14 ± 0.08	0.70 ± 0.03	0.56 ± 0.05	Cu	0.50 ± 0.01	0.26 ± 0.01	0.38 ± 0.002	0.47 ± 0.01
Mn	18.80 ± 0.01	28.90 ± 0.004	12.20 ± 0.01	0.01 ± 0.01	Mn	26.20 ± 0.01	19.202 ± 0.004	6.22 ± 0.03	0.55 ± 0.01
Ni	0.15 ± 0.03	0.04 ± 0.05	1.76 ± 0.01	0.73 ± 0.05	Ni	0.47 ± 0.01	0.01 ± 0.02	1.84 ± 0.004	0.28 ± 0.01
Pb	0.14 ± 0.03	0.20 ± 0.02	0.50 ± 0.02	BDL	Pb	0.23 ± 0.02	0.01 ± 0.10	0.55 ± 0.01	0.23 ± 0.03
V	0.15 ± 0.03	0.11 ± 0.02	0.01 ± 0.02	0.40 ± 0.02	V	0.20 ± 0.02	0.15 ± 0.01	0.01 ± 0.19	0.25 ± 0.04
Zn	0.27 ± 0.01	0.47 ± 0.002	1.01 ± 0.02	6.47 ± 0.01	Zn	0.33 ± 0.01	0.54 ± 0.01	0.48 ± 0.02	0.68 ± 0.01
**MHT**					**KFT**				
Cd	BDL	BDL	1.28 ± 0.01	0.26 ± 0.004	Cd	0.44 ± 0.02	0.02 ± 0.14	1.50 ± 0.20	0.02 ± 0.23
Cr	0.36 ± 0.003	0.06 ± 0.03	0.81 ± 0.002	0.78 ± 0.01	Cr	0.29 ± 0.01	0.04 ± 0.04	0.98 ± 0.002	1.17 ± 0.04
Cu	0.59 ± 0.01	0.25 ± 0.03	0.21 ± 0.01	0.39 ± 0.03	Cu	0.57 ± 0.02	0.25 ± 0.05	0.41 ± 0.02	0.73 ± 0.02
Mn	1.38 ± 0.002	0.46 ± 0.03	0.28 ± 0.01	0.44 ± 0.01	Mn	14.55 ± 0.02	8.49 ± 0.01	1.66 ± 0.02	0.63 ± 0.01
Ni	0.26 ± 0.01	BDL	1.61 ± 0.01	0.30 ± 0.02	Ni	0.41 ± 0.01	BDL	1.85 ± 0.01	0.26 ± 0.02
Pb	0.04 ± 0.11	BDL	0.66 ± 0.01	0.15 ± 0.01	Pb	0.13 ± 0.02	0.02 ± 0.07	0.67 ± 0.01	0.06 ± 0.01
V	0.34 ± 0.02	0.16 ± 0.01	0.03 ± 0.08	0.33 ± 0.03	V	0.19 ± 0.02	0.16 ± 0.01	0.02 ± 0.05	0.43 ± 0.06
Zn	0.77 ± 0.01	0.31 ± 0.02	1.58 ± 0.02	0.52 ± 0.01	Zn	0.71 ± 0.01	0.58 ± 0.02	0.45 ± 0.02	0.27 ± 0.02
**SSH**					**ACT**				
Cd	0.15 ± 0.25	BDL	1.30 ± .03	0.50 ± 0.01	Cd	0.73 ± 0.03	0.33 ± 0.05	1.60 ± 0.01	0.51 ± 0.05
Cr	0.26 ± 0.002	0.04 ± 0.05	0.86 ± 0.004	1.29 ± 0.002	Cr	0.29 ± 0.01	0.04 ± 0.04	0.96 ± 0.001	0.96 ± 0.001
Cu	0.37 ± 0.02	0.24 ± 0.05	0.14 ± 0.02	0.64 ± 0.01	Cu	0.49 ± 0.01	0.26 ± 0.05	0.35 ± 0.01	0.35 ± 0.05
Mn	1.89 ± 0.02	1.10 ± 0.07	0.24 ± 0.01	0.26 ± 0.02	Mn	30.1 ± 0.02	22.1 ± 0.01	6.52 ± 0.02	0.97 ± 0.004
Ni	0.27 ± 0.01	BDL	1.66 ± 0.01	0.58 ± 0.002	Ni	0.43 ± 0.01	0.05 ± 0.02	1.84 ± 0.01	0.51 ± 0.003
Pb	0.05 ± 0.05	BDL	0.52 ± 0.01	0.16 ± 0.03	Pb	0.76 ± 0.03	0.13 ± 0.01	0.54 ± 0.01	0.32 ± 0.03
V	0.17 ± 0.01	0.14 ± 0.04	0.02 ± 0.10	0.24 ± 0.04	V	0.20 ± 0.03	0.14 ± 0.02	BDL	0.19 ± 0.01
Zn	0.31 ± 0.03	0.27 ± 0.01	0.44 ± 0.02	0.39 ± 0.02	Zn	0.33 ± 0.01	0.34 ± 0.003	0.45 ± 0.04	0.74 ± 0.01

BDL = Below detection limit

**Table 4:** Concentration (mean ± S.D) (mg/kg) of Metals from Sequential Extraction of Green Tea Samples (n = 11).

**TPG**	F1	F2	F3	F4	**LGL**	F1	F2	F3	F4
Cd	1.11 ± 0.01	0.18 ± 0.04	1.34 ± 0.01	0.65 ± 0.04	Cd	1.16 ± 0.03	0.14 ± 0.01	1.46 ± 0.02	0.05 ± 0.15
Cr	0.17 ± 0.02	0.03 ± 0.03	0.75 ± 0.003	0.45 ± 0.001	Cr	0.16 ± 0.01	0.06 ± 0.03	0.79 ± 0.01	0.11 ± 0.004
Cu	0.09 ± 0.07	0.18 ± 0.05	0.27 ± 0.01	0.76 ± 0.04	Cu	0.10 ± 0.02	0.24 ± 0.02	0.23 ± 0.01	0.22 ± 0.05
Mn	19.50 ± 0.002	10.40 ± 0.01	1.19 ± 0.001	0.79 ± 0.01	Mn	20.10 ± 0.01	11.60 ± 0.02	2.15 ± 0.01	0.13 ± 0.02
Ni	0.44 ± 0.01	0.06 ± 0.04	1.52 ± 0.04	0.61 ± 0.03	Ni	0.65 ± 0.01	0.05 ± 0.02	0.16 ± 0.01	0.02 ± 0.02
Pb	0.51 ± 0.01	0.07 ± 0.03	0.51 ± 0.05	0.24 ± 0.02	Pb	0.36 ± 0.01	0.05 ± 0.09	0.52 ± 0.00	0.07 ± 0.04
V	0.04 ± 0.003	0.13 ± 0.02	0.03 ± 0.01	0.26 ± 0.03	V	0.01 ± 0.001	0.13 ± 0.01	0.05 ± 0.10	0.01 ± 0.17
Zn	1.99 ± 0.01	0.45 ± 0.01	0.16 ± 0.04	0.89 ± 0.01	Zn	0.39 ± 0.01	0.30 ± 0.02	0.25 ± 0.03	0.76 ± 0.02
**HGT**					**LGR**				
Cd	1.08 ± 0.02	0.24 ± 0.001	1.77 ± 0.01	1.35 ± 0.03	Cd	1.00 ± 0.20	0.14 ± 0.03	1.42 ± 0.01	0.66 ± 0.04
Cr	0.11 ± 0.05	0.02 ± 0.001	0.82 ± 0.01	1.35 ± 0.004	Cr	0.23 ± 0.02	0.06 ± 0.01	0.83 ± 0.003	0.79 ± 0.02
Cu	0.05 ± 0.11	0.13 ± 0.04	0.94 ± 0.004	0.25 ± 0.05	Cu	0.18 ± 0.03	0.27 ± 0.04	0.25 ± 0.03	0.34 ± 0.05
Mn	15.9 ± 0.01	13.6 ± 0.01	6.23 ± 0.001	0.96 ± 0.003	Mn	20.4 ± 0.01	10.8 ± 0.01	1.43 ± 0.01	BDL
Ni	0.48 ± 0.01	0.02 ± 0.08	1.83 ± 0.003	1.33 ± 0.01	Ni	0.67 ± 0.01	0.04 ± 0.07	1.66 ± 0.01	0.65 ± 0.03
Pb	0.44 ± 0.02	0.09 ± 0.03	0.55 ± 0.02	0.73 ± 0.02	Pb	0.31 ± 0.01	0.04 ± 0.07	0.30 ± 0.02	0.13 ± 0.38
V	0.01 ± 0.001	0.10 ± 0.01	0.07 ± 0.06	BDL	V	0.04 ± 0.13	0.14 ± 0.04	0.03 ± 0.08	0.20 ± 0.04
Zn	0.65 ± 0.01	0.44 ± 0.02	0.75 ± 0.01	8.37 ± 0.03	Zn	0.33 ± 0.003	0.24 ± 0.01	0.17 ± 0.02	2.47 ± 0.02
**GBG**					**LGJ**				
Cd	0.99 ± 0.04	0.13 ± 0.06	1.44 ± 0.01	1.59 ± 0.01	Cd	0.27 ± 0.05	BDL	1.22 ± 0.02	0.80 ± 0.04
Cr	0.01 ± 0.001	0.03 ± 0.02	0.69 ± 0.01	1.31 ± 0.01	Cr	0.17 ± 0.02	0.05 ± 0.04	0.82 ± 0.01	1.51 ± 0.002
Cu	BDL	0.17 ± 0.05	0.32 ± 0.01	0.49 ± 0.02	Cu	0.26 ± 0.04	0.25 ± 0.02	0.14 ± 0.01	0.23 ± 0.04
Mn	26.60 ± 0.002	10.70 ± 0.01	2.61 ± 0.01	0.50 ± 0.01	Mn	7.05 ± 0.01	4.41 ± 0.02	0.20 ± 0.01	0.09 ± 0.02
Ni	0.23 ± 0.002	0.03 ± 0.04	1.48 ± 0.01	1.51 ± 0.01	Ni	0.30 ± 0.01	0.03 ± 0.07	1.57 ± 0.01	0.79 ± 0.01
Pb	0.39 ± 0.02	0.08 ± 0.05	0.44 ± 0.01	0.75 ± 0.002	Pb	0.09 ± 0.03	BDL	0.51 ± 0.01	0.40 ± 0.01
V	0.03 ± 0.003	0.11 ± 0.03	0.07 ± 0.02	0.02 ± 0.02	V	0.11 ± 0.04	0.15 ± 0.02	0.03 ± 0.09	BDL
Zn	0.31 ± 0.02	0.56 ± 0.01	0.28 ± 0.03	2.32 ± 0.01	Zn	0.18 ± 0.02	0.25 ± 0.01	0.08 ± 0.03	4.21 ± 0.01
**SBG**					**LGS**				
Cd	0.83 ± 0.4	0.06 ± 0.08	1.50 ± 0.02	0.71 ± 0.04	Cd	1.03 ± 0.01	0.27 ± 0.01	1.60 ± 0.01	1.29 ± 0.01
Cr	0.16 ± 0.02	0.06 ± 0.01	0.79 ± 0.01	0.86 ± 0.002	Cr	0.13 ± 0.01	0.04 ± 0.01	0.890 ± 0.004	1.13 ± 0.001
Cu	0.03 ± 0.03	0.18 ± 0.03	0.30 ± 0.01	0.65 ± 0.02	Cu	0.26 ± 0.05	0.31 ± 0.05	0.38 ± 0.02	0.02 ± 0.11
Mn	9.16 ± 0.01	4.94 ± 0.01	0.93 ± 0.02	0.22 ± 0.01	Mn	33.0 ± 0.01	21.90 ± 0.01	5.71 ± 0.01	1.28 ± 0.01
Ni	0.54 ± 0.003	0.05 ± 0.04	1.67 ± 0.01	0.71 ± 0.003	Ni	0.45 ± 0.01	0.03 ± 0.05	1.78 ± 0.01	1.23 ± 0.01
Pb	0.23 ± 0.03	0.03 ± 0.12	0.30 ± 0.003	0.25 ± 0.02	Pb	0.40 ± 0.03	0.09 ± 0.01	0.54 ± 0.01	0.62 ± 0.01
V	0.02 ± 0.002	0.11 ± 0.05	0.06 ± 0.01	0.10 ± 0.001	V	0.06 ± 0.08	0.18 ± 0.03	0.03 ± 0.05	0.04 ± 0.001
Zn	0.58 ± 0.01	0.40 ± 0.01	0.21 ± 0.04	0.17 ± 0.03	Zn	0.35 ± 0.01	0.35 ± 0.02	0.38 ± 0.02	24.4 ± 0.01
**LBG**					**BGT**				
Cd	1.47 ± 0.01	0.15 ± 0.04	1.72 ± 0.02	1.45 ± 0.03	Cd	0.98 ± 0.02	0.34 ± 0.04	1.57 ± 0.02	0.37 ± 0.04
Cr	0.13 ± 0.03	0.07 ± 0.01	0.82 ± 0.01	1.53 ± 0.004	Cr	0.18 ± 0.01	BDL	0.93 ± 0.01	1.06 ± 0.01
Cu	0.64 ± 0.004	0.21 ± 0.04	0.30 ± 0.01	0.60 ± 0.02	Cu	0.23 ± 0.03	0.14 ± 0.05	0.340 ± 0.004	0.77 ± 0.03
Mn	27.40 ± 0.01	7.89 ± 0.01	1.18 ± 0.01	0.21 ± 0.003	Mn	30.10 ± 0.01	19.20 ± 0.02	3.39 ± 0.77	1.45 ± 0.02
Ni	0.69 ± 0.004	0.07 ± 0.02	1.94 ± 0.02	1.57 ± 0.004	Ni	0.44 ± 0.02	0.05 ± 0.04	1.81 ± 0.01	0.36 ± 0.01
Pb	0.49 ± 0.02	0.05 ± 0.03	0.43 ± 0.01	1.24 ± 0.01	Pb	0.37 ± 0.03	0.14 ± 0.02	0.63 ± 0.02	0.78 ± 0.02
V	BDL	0.13 ± 0.03	0.06 ± 0.01	0.09 ± 0.04	V	0.08 ± 0.04	0.12 ± 0.05	0.02 ± 0.16	0.47 ± 0.03
Zn	0.69 ± 0.01	0.33 ± 0.02	0.43 ± 0.02	6.09 ± 0.01	Zn	0.34 ± 0.02	0.39 ± 0.02	0.28 ± 0.02	5.09 ± 0.01
					**TWG**				
					Cd	0.73 ± 0.02	0.15 ± 0.18	1.44 ± 0.04	0.39 ± 0.02
					Cr	0.33 ± 0.00	0.037 ± 0.00	0.85 ± 0.01	1.09 ± 0.01
					Cu	0.44 ± 0.02	0.25 ± 0.01	0.30 ± 0.01	0.46 ± 0.02
					Mn	20.90 ± 0.01	14.20 ± 0.01	2.59 ± 0.001	1.48 ± 0.01
					Ni	0.49 ± 0.00	0.02 ± 0.09	1.65 ± 0.01	0.440 ± 0.002
					Pb	0.21 ± 0.03	0.05 ± 0.02	0.52 ± 0.01	0.33 ± 0.01
					V	0.16 ± 0.03	0.15 ± 0.02	0.02 ± 0.25	0.57 ± 0.02
					Zn	0.36 ± 0.01	0.32 ± 0.04	0.24 ± 0.03	3.92 ± 0.02

**Table 5:** Concentration (mean ± S.D) (mg/kg) of Metals from Sequential Extraction of Black Tea Samples (n = 4).

	F1	F2	F3	F4		F1	F2	F3	F4
**LYL**					**TTL**				
Cd	0.62 ± 0.05	0.28 ± 0.04	1.56 ± 0.02	0.57 ± 0.01	Cd	0.60 ± 0.03	0.12 ± 0.03	1.57 ± 0.01	0.78 ± 0.02
Cr	0.25 ± 0.01	BDL	0.93 ± 0.01	0.69 ± 0.01	Cr	0.41 ± 0.003	0.03 ± 0.02	0.99 ± 0.01	1.17 ± 0.001
Cu	0.38 ± 0.01	0.15 ± 0.04	0.40 ± 0.02	0.42 ± 0.04	Cu	0.47 ± 0.01	0.26 ± 0.02	0.35 ± 0.01	0.27 ± 0.02
Mn	23.90 ± 0.01	18.80 ± 0.01	3.50 ± 0.001	1.79 ± 0.02	Mn	18.70 ± 0.02	15.7 ± 0.01	2.33 ± 0.02	0.30 ± 0.001
Ni	0.30 ± 0.01	0.03 ± 0.03	1.87 ± 0.01	0.66 ± 0.01	Ni	0.55 ± 0.01	0.01 ± 0.08	1.90 ± 0.01	0.77 ± 0.01
Pb	0.18 ± 0.02	0.12 ± 0.04	0.56 ± 0.02	0.26 ± 0.02	Pb	0.21 ± 0.04	0.06 ± 0.03	0.61 ± 0.02	0.34 ± 0.04
V	0.15 ± 0.02	0.10 ± 0.02	0.01 ± 0.003	0.45 ± 0.02	V	0.23 ± 0.02	0.16 ± 0.04	0.01 ± 0.07	0.12 ± 0.02
Zn	0.48 ± 0.01	0.27 ± 0.002	0.20 ± 0.04	1.30 ± 0.01	Zn	0.30 ± 0.04	0.26 ± 0.03	0.35 ± 0.04	0.21 ± 0.04
**TTG**					**TTR**				
Cd	0.64 ± 0.02	0.29 ± 0.003	1.53 ± 0.02	0.59 ± 0.03	Cd	0.54 ± 0.03	0.16 ± 0.05	1.53 ± 0.02	0.58 ± 0.04
Cr	0.30 ± 0.01	0.01 ± 0.05	0.91 ± 0.01	0.96 ± 0.01	Cr	0.28 ± 0.01	0.02 ± 0.01	0.93 ± 0.01	1.25 ± 0.01
Cu	0.35 ± 0.02	0.19 ± 0.08	0.37 ± 0.01	0.53 ± 0.01	Cu	0.41 ± 0.003	0.26 ± 0.02	0.36 ± 0.01	0.35 ± 0.03
Mn	21.60 ± 0.01	20.90 ± 0.01	4.10 ± 0.02	1.73 ± 0.01	Mn	19.40 ± 0.01	17.70 ± 0.01	3.14 ± 0.01	0.22 ± 0.01
Ni	0.36 ± 0.03	0.03 ± 0.05	1.81 ± 0.01	0.64 ± 0.01	Ni	0.35 ± 0.004	0.02 ± 0.05	1.08 ± 0.003	0.57 ± 0.01
Pb	0.20 ± 0.02	0.11 ± 0.05	0.52 ± 0.01	0.36 ± 0.03	Pb	0.15 ± 0.01	0.08 ± 0.02	0.52 ± 0.01	0.27 ± 0.01
V	0.16 ± 0.02	0.12 ± 0.04	0.01 ± 0.09	0.35 ± 0.04	V	0.19 ± 0.01	0.16 ± 0.03	0.02 ± 0.06	0.17 ± 0.02
Zn	0.33 ± 0.01	0.36 ± 0.01	0.50 ± 0.02	6.23 ± 0.01	Zn	0.30 ± 0.003	0.33 ± 0.01	0.37 ± 0.01	0.33 ± 0.03

**Table 6:** Pseudo-total Trace Metal Concentrations (mg/kg) (mean ± S.D) in Green, Herbal and Black Tea Samples.

	**Cd**		**Cr**	**Cu**	**Mn**	**Ni**	**Pb**	**V**	**Zn**
**Green Tea**									
TPG	0.22 ± 0.004		0.06 ± 0.003	0.19 ± 0.004	4.90 ± 0.003	0.10 ± 0.001	0.07 ± 0.003	0.02 ± 0.002	0.65 ± 0.001
HGT	0.43 ± 0.03		0.09 ± 0.003	0.31 ± 0.01	8.26 ± 0.33	0.16 ± 0.10	0.13 ± 0.001	0.03 ± 0.001	0.74 ± 0.04
GBG	0.59 ± 0.003		0.08 ± 0.00	0.39 ± 0.01	13.40 ± 0.02	0.18 ± 0.01	0.16 ± 0.003	0.05 ± 0.001	0.90 ± 0.01
SBG	0.32 ± 0.08		0.10 ± 0.04	0.22 ± 0.04	4.55 ± 0.22	0.15 ± 0.05	0.07 ± 0.01	0.03 ± 0.02	0.79 ± 0.13
LBG	0.43 ± 0.01		0.11 ± 0.004	0.18 ± 0.001	8.13 ± 0.01	0.17 ± 0.003	0.09 ± 0.004	0.04 ± 0.004	0.93 ± 0.09
LGL	0.57 ± 0.19		0.13 ± 0.04	0.17 ± 0.01	6.77 ± 3.91	0.40 ± 0.37	0.11 ± 0.03	0.03 ± 0.01	0.63 ± 0.11
LGR	0.44 ± 0.01		0.12 ± 0.002	0.14 ± 0.001	9.61 ± 0.14	0.18 ± 0.01	0.09 ± 0.01	0.03 ± 0.001	0.28 ± 0.01
LGJ	0.29 ± 0.001		0.10 ± 0.001	0.13 ± 0.002	5.36 ± 0.01	0.14 ± 0.003	0.06 ± 0.001	0.04 ± 0.004	0.29 ± 0.05
LGS	0.53 ± 0.01		0.10 ± 0.003	0.19 ± 0.002	14.70 ± 0.06	0.18 ± 0.002	0.16 ± 0.002	0.03 ± 0.003	0.34 ± 0.002
BGT	0.59 ± 0.08		0.18 ± 0.02	0.17 ± 0.001	13.30 ± 0.08	0.25 ± 0.06	0.14 ± 0.01	0.03 ± 0.01	0.38 ± 0.04
TWG	0.51 ± 0.04		0.14 ± 0.01	0.17 ± 0.004	11.30 ± 0.07	0.21 ± 0.02	0.11 ± 0.01	0.03 ± 0.00	0.37 ± 0.08
**Herbal Tea**									
NLF	0.64 ± 0.001	0.18 ± 0.001		0.18 ± 0.00	13.70 ± 0.02	0.42 ± 0.002	0.12 ± 0.001	0.01 ± 0.001	0.46 ± 0.01
TBN	0.68 ± 0.08	2.51 ± 2.02		0.30 ± 0.12	17.30 ± 3.70	0.29 ± 0.04	0.20 ± 0.06	0.02 ± 0.001	0.60 ± 0.17
MHT	0.72 ± 0.003	0.20 ± 0.001		0.14 ± 0.00	0.89 ± 0.01	0.89 ± 0.001	0.13 ± 0.002	0.02 ± 0.003	0.73 ± 0.01
SSH	0.92 ± 0.01	0.50 ± 0.01		0.22 ± 0.00	1.26 ± 0.08	1.10 ± 0.01	0.07 ± 0.001	0.03 ± 0.001	0.48 ± 0.08
AHT	0.55 ± 0.03	0.13 ± 0.001		0.25 ± 0.00	11.9 ± 0.36	0.30 ± 0.001	0.11 ± 0.001	0.01 ± 0.001	0.55 ± 0.001
JCT	0.54 ± 0.01	0.11 ± 0.003		0.19 ± 0.00	14.8 ± 0.19	0.19 ± 0.004	0.19 ± 0.004	0.02 ± 0.01	0.55 ± 0.001
KFT	0.44 ± 0.01	0.19 ± 0.001		0.33 ± 0.00	7.91 ± 0.04	0.22 ± 0.001	0.11 ± 0.002	0.03 ± 0.01	0.73 ± 0.01
ACT	0.56 ± 0.004	0.11 ± 0.002		0.20 ± 0.00	17.2 ± 0.002	0.18 ± 0.003	0.18 ± 0.001	0.02 ± 0.002	0.42 ± 0.003
**Black Tea**									
LYL	0.36 ± 0.02	0.39 ± 0.01		0.15 ± 0.003	10.2 ± 0.09	0.17 ± 0.003	0.08 ± 0.003	0.02 ± 0.003	0.30 ± 0.06
TTG	0.71 ± 0.01	0.30 ± 0.004		0.31 ± 0.001	8.47 ± 0.10	0.54 ± 0.01	0.12 ± 0.001	0.01 ± 0.003	0.55 ± 0.01
TTL	0.42 ± 0.001	0.16 ± 0.001		0.15 ± 0.002	11.4 ± 0.05	0.17 ± 0.001	0.12 ± 0.001	0.03 ± 0.01	0.29 ± 0.02
TTR	0.53 ± 0.001	0.27 ± 0.01		0.19 ± 0.001	12.4 ± 0.09	0.19 ± 0.001	0.17 ± 0.002	0.13 ± 0.002	0.42 ± 0.03

## 2 Experimental design, materials and methods

### 2.1 Sample collection and preparation

Twenty-three (23) tea samples (including 11 green, 8 herbal and 4 black tea brands) were purchased from supermarkets in Lagos and Ogun states, Nigeria. The manufacturers’ information for each brand of tea was recorded. Samples were weighed and oven-dried at 105 °C for 30 min. Sample size was reduced and homogenized by coning and quartering, followed by milling in a clean porcelain mortar before sequential and pseudo-total extraction procedure (Table 2). Samples meant for PAHs analyses were not subjected to oven drying procedure.

### 2.2 Sequential extraction

See Table 2.

### 2.3 Acid digestion for pseudo-total elemental contents

Aqua regia was prepared in a 3:1 ratio of concentrated HCl and HNO_3_. Then 1.0 g of the dry ground tea sample was weighed into a 250 mL conical flask. Fifty (50) mL of aqua regia was added to the sample and the conical flask was transferred to a Stuart heat-stir hot plate to heat at controlled temperatures between 50 °C and 250 °C for about 2 hours in the fume cupboard. Additional twenty (20) mL of aqua regia was added as the volume reduced until a clear solution was obtained. The cold solution was filtered and made up to 100 mL with deionized water in a 100 mL volumetric flask and taken to the MP-AES for analysis.

### 2.4 Calibration curves

The standard stock solution (1000 mg/L BDH grade) was appropriately diluted to prepare the calibration standards of each heavy metal determined. Stock solutions of 0.5, 1.0 and 2.0 mg/L of Cd, Cr, Cu, Mn, Ni, Pb, V and Zn were used to establish the calibration curves. The limit of detection (LOD) and limit of quantitation (LOQ) were determined using data from the calibration curves as 3 and 10 times the standard deviation of the standards, respectively (Table 7). Recovery study was carried out for an assessment of the analytical measurement procedure.

**Table 7:** Parameters used to establish the calibration curves and the emission wavelengths for metals.

	**R**^**2**^	**Linear equation**	**LOD (mg/kg)**	**LOQ (mg/kg)**	**Wavelength, λ (nm)**
Cd	0.9999	*Y* = 445.2896*C* + 6.4903	0.001	0.01	361.051
Cr	0.9998	*Y* = 9025.1435*C* + 9.7799	0.001	0.004	357.868
Cu	0.9997	*Y* = 41816.0982*C* + 1.2289	0.001	0.002	324.754
Mn	0.9996	*Y* = 10737.4557*C* +28.9041	0.001	0.01	259.372
Ni	0.9997	*Y* = 5378.0546*C* + 40.0364	0.01	0.005	352.454
Pb	0.9998	*Y* = 1266.01515*C* + 12.4557	0.01	0.02	405.781
V	0.9998	*Y* = 1513.3939*C* + 8.6272	0.001	0.02	318.539
Zn	0.9990	*Y* = 4323.2681*C* +29.9143	0.008	0.02	213.857

R^2^ = Correlation coefficient

### 2.5 Instrumentation for PAHs analysis

PAHs were analyzed using an Agilent 7890A with an auto-sampler Agilent 7683B, coupled to flame ionization detector (FID). The GC is equipped with an HP-5 column (19091J-413) (30 m × 0.32 mm × 0.25 µm) from Agilent (USA). The carrier gas used was helium maintained at a flow rate of 4.84 mL/min. The oven temperature program is as follows: 0.4 min at 50 °C, to 195 °C at 20 °C/min, hold 3.0 min, to 250 °C at 8 °C/min, hold 5.0 min, to 290 °C at 5 °C/min, hold 1.0 min. Helium and nitrogen gases with 99.9999% purity were purchased from Foshan Huate Gas Coy Ltd. (China). US-EPA 16 priority PAHs (Acenaphthene, ACN, acenaphthylene, ACY, anthracene, ANT, benzo(a)anthracene, BaA, benzo(a)pyrene, BaP, benzo(b)fluoranthene, BbF, benzo(g,h,i)perylene, BghiP, dibenzo(a,h)anthracene, DahA, fluoranthene, FLA, benzo(k)fluoranthene, BkF, chrysene, CHR, indeno(1,2,3cd)pyrene, IP, phenanthrene, PHE, naphthalene, NAP, fluorene, FLR, and pyrene, PYR) were considered in the present study. The standard PAH calibration mix used was a 2.0 mg/mL stock solution in dichloromethane:benzene (1:1) (AccuStandard No. Z-014G-R) with individual PAH concentrations: ACN 2002 ± 0.4 µg/mL, ACY 1984 ± 2.1 µg/mL, ANT 1999 ± 3.2 µg/mL, BaA 2003 ± 14.4 µg/mL, BaP 2007 ± 17.1 µg/mL, BbF 2004 ± 1.3 µg/mL, BghiP 1982 ± 4.3 µg/mL, BkF 1987 ± 14.5 µg/mL, CHR 2005 ± 0.8 µg/mL, DahA 1981 ± 4.6 µg/mL, FLA 2000 ± 3.5 µg/mL, FLR 1966 ± 10.4 µg/mL, IP 1997 ± 4.5 µg/mL, NAP 1995 ± 3.4 µg/mL, PHE 2004 ± 0.1 µg/mL, PYR 1983 ± 2.1 µg/mL, and CBZ 1994 ± 2.2 µg/mL. Triplicate determinations were made on all extracted tea samples. Recovery study was carried out for an assessment of the measurement procedure. The recoveries of each individual PAH varied from 90.24 to 108.92% for PHE and DahA, respectively.
